# A three-year-old boy with X-linked adrenoleukodystrophy and congenital pulmonary adenomatoid malformation: a case report

**DOI:** 10.1186/1752-1947-3-9329

**Published:** 2009-12-14

**Authors:** Ibrahim Abdulhamid, Sermin Saadeh, Nedim Cakan

**Affiliations:** 1 Pediatric Pulmonary Division, The Carman and Ann Adams Department of Pediatrics, Wayne State University, Children's Hospital of Michigan. 3901 Beaubien BLVD, Detroit, MI 48201, USA; 2 Pediatric Residency Program, The Carman and Ann Adams Department of Pediatrics, Wayne State University, Children's Hospital of Michigan. 3901 Beaubien BLVD, Detroit, MI 48201, USA; 3 Pediatric Endocrine Division, The Carman and Ann Adams Department of Pediatrics, Wayne State University, Children's Hospital of Michigan. 3901 Beaubien BLVD, Detroit, MI 48201, USA

## Abstract

**Introduction:**

X-linked adrenoleukodystrophy leads to demyelination of the nervous system, adrenal insufficiency, and accumulation of long-chain fatty acids. Most young patients with X-linked adrenoleukodystrophy develop seizures and progressive neurologic deficits, and die within the first two decades of life. Congenital or acquired disorders of the respiratory system have not been previously described in patients with X-linked adrenoleukodystrophy.

**Case presentation:**

A 3-year-old Arabic boy from Yemen presented with discoloration of the mucous membranes and nail beds, which were considered cyanoses due to methemoglobinemia. He also had shortness of breath, fatigue, emesis and dehydration episodes for which he was admitted to our hospital. Chest radiograph and chest computed tomography scans showed congenital pulmonary adenomatoid malformation. A few weeks before the removal of the malformation, he had a significant episode of hypotension and hypoglycemia. This development required further in-hospital evaluation that led to the diagnosis of adrenal insufficiency and the initiation of treatment with corticosteroids. One year later, he developed seizures and loss of consciousness. Magnetic resonance imaging of his head showed diffuse demyelination secondary to X-linked adrenoleukodystrophy. He was treated with anti-seizure and anti-oxidants, and was referred for bone marrow transplant evaluation.

**Conclusion:**

The presence of adrenal insufficiency, neurologic deficits and seizures are common manifestations of X-linked adrenoleukodystrophy. The association of congenital lung disease with X-linked adrenoleukodystrophy or Addison's disease has not been described previously.

## Introduction

X-linked adrenoleukodystrophy (X-ALD) is the most common inherited peroxisomal disorder with an incidence of 1:20,000 males [1,2]. It is caused by defects of the ABCD1 gene on chromosome Xq28 [1,2]. X-ALD leads to the impairment of peroxisomal β-oxidation, accumulation of very long chain fatty acids (VLCFA), progressive demyelination of the nervous system, and adrenal insufficiency [1,2]. The phenotypic presentations are highly variable, which may lead to delayed recognition and misdiagnosis as attention and/or hyperactivity deficit disorder in boys or multiple sclerosis in adults [1,2].

Hydrocortisone and mineralocorticosteroids are necessary to treat adrenal insufficiency. High doses of hydrocortisone preoperatively and during recovery are needed for surgery and other stressful illnesses in affected individuals [3].

Congenital pulmonary adenomatoid malformation (CPAM) is the most common cystic lung lesion diagnosed pre- and post-natally [4]. CPAM may lead to respiratory distress, recurrent lung infections and pulmonary tumors if not surgically excised [4]. In this report, we describe a preschool boy who presented with intermittent symptoms of respiratory distress, lethargy, dehydration, hypoglycemia, hypotension, hyperpigmentation and large CPAM. He was eventually diagnosed with adrenal insufficiency. He had a surgical removal of the CPAM to relieve his respiratory distress symptoms, prevent infections, and thwart cancerous transformation. Subsequently, he developed seizures and neurologic symptoms and was diagnosed with X-ALD after further evaluation.

The association of X-ALD or adrenal insufficiency with congenital respiratory lesions was not previously reported in the pediatric age group.

## Case presentation

A 3-year-old Arabic boy from Yemen presented with intermittent episodes of shortness of breath, lethargy, fatigability, vomiting, and "cyanotic" discoloration of his skin, lips, mucous membranes and nail beds for the past two years. He was thought to have methemoglobinemia due to persistent cyanosis. He was born in Yemen and moved to the USA for further treatment one year prior to his visit to our clinic. He was evaluated in several institutions in Yemen, Europe and the USA for these problems but no specific diagnosis was ever made. He had one maternal uncle who died suddenly at 35 years of age and 4 full siblings (all males) who died of unknown causes between 2 and 4 years of age. His deceased siblings had similar symptoms and discoloration of mucous membranes. None of them had autopsies. He had 4 other siblings (2 brothers and 2 sisters) who were alive with no medical problems. He had normal electrolytes, blood urea nitrogen (BUN) and creatinine (Cr) levels on several occasions. He had normal results for cardiac examination, echocardiogram, hematologic evaluation and hemoglobin electrophoresis.

**Figure 1:**
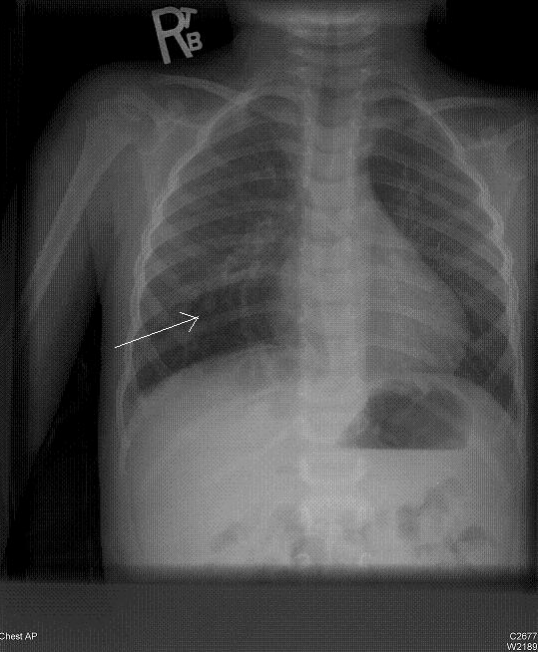
**Posteroanterior view of the chest showing hyperlucency in the right lower lobe area (arrow)**.

**Figure 2:**
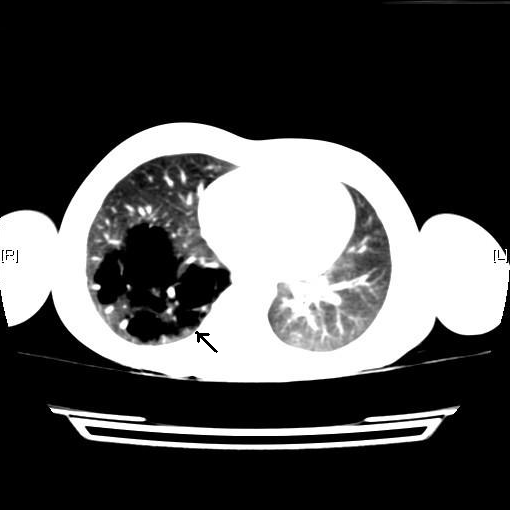
**Computed tomography scan of the chest showing multiple cystic lesions of congenital pulmonary adenomatoid malformation in the right lower lobe (arrow)**.

He was referred to our center for further evaluation of his cyanosis. The result of his physical examination was normal, except for the noted discoloration of his lips and nail beds. His arterial blood showed mild hypoxemia as follows: pH 7.32, PaCO2 37.1 mmHg, PaO2 86 mmHg, and HCO3 18.9. His methemoglobin and carboxyhemoglobin levels were 1.10% and 0.3%, respectively. He had a previous chest X-ray during one of his prior admissions which was interpreted as normal. However, upon further examination of the film at our clinic, we identified a subtle parenchymal hyperlucency of a large part of his right mid-lung area (Figure 1). His chest computed tomography (CT) scan showed multiple cystic lesions in his right lung that was compatible with CPAM (Figure 2). CT scan of the abdomen showed no abnormalities of the adrenal glands, or other abdominal organs.

Before the surgical removal of his CPAM, he was admitted with lethargy, vomiting, dehydration, hypotension, and drowsiness. Laboratory results during this hospitalization were as follows: glucose, 2.42 mmol/L (44 mg/dl); sodium, 132 mmol/L; potassuim 3.8 mmol/L; chloride, 101 mmol/L; bicarbonate, 13 mmol/L; BUN, 7.5 mmol/L (21 mg/dL); Cr, 17.68 umol/L (0.2 mg/dL); calcuim, 2.17 mmol/L (8.7 mg/dL); magnesium, 0.57 mmol/L (1.4 mg/dL); and phosphorous, 1.2 mmol/L (3.7 mg/dL).

**Figure 3:**
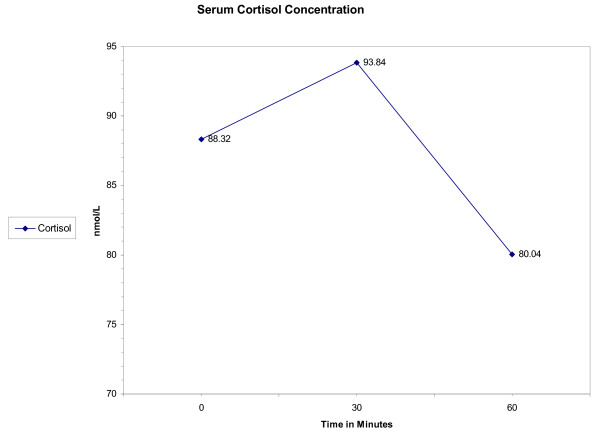
Cortisol levels before and after adrenocorticotropic hormone stimulation

After the initial resuscitation with boluses of 25% dextrose and normal saline solutions, a repeat glucose test showed a value of 21.78 mmol/L (396 mg/dL). One hour after that, his glucose level dropped again to 2.1 mmol/L (39 mg/dl), and a second 25% dextrose solution bolus was thus given. Our patient had low serum cortisol level, normal aldosterone, and normal growth hormone concentrations (Table 1). His serum adrenocorticotropic hormone (ACTH) concentration was elevated at 2,630 pg/ml. ACTH stimulation test did not result in an increase in his cortisol levels (Figure 3). He had no detectable anti-adrenal antibodies and a non-reactive purified protein derivative skin test. He was started on hydrocortisone and fludrocortisone and had a surgical removal of the CPAM a few weeks later.

**Table 1:** Hormone concentration

Hormone	Serum concentration
ACTH	**584.386 pmol/L**

Fasting cortisol	**79.999 nmol/L**

Aldosterone	**110.988 pmol/L**

Growth hormone	**4.4 ug/L**

Insulin	**<13.89 pmol/L**

Our patient's serum ACTH concentration decreased to 13 pg/ml six months after the treatment. A pathological examination of the lung cysts showed multiple thin-walled cysts that ranged from 0.3 cm to 1.5 cm in diameter and filled with clear fluid. The cysts appeared to occupy approximately 90% of our patient's parenchyma. Microscopically, the cysts were lined with columnar (respiratory type) epithelium. This was compatible with the diagnosis of CPAM.

**Figure 4:**
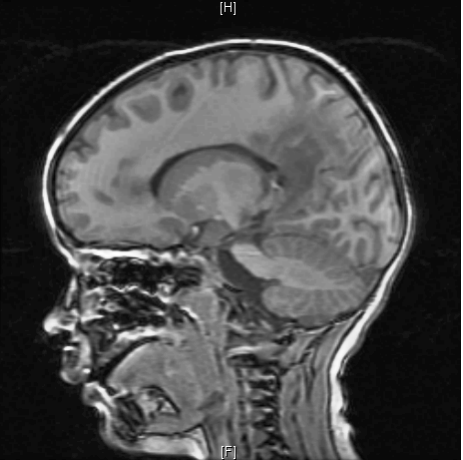
Magnetic resonance imaging of the brain showing bilateral symmetric demyelination of various parts of the brain

One year later, he was readmitted with a seizure and loss of consciousness. A brain magnetic resonance imaging (MRI) revealed bilaterally diffuse symmetric high T2 and FLAIR signal abnormality involving the white matter of several parts of his brain, which was suggestive of a diffuse and active demyelination process (Figures 4). He had elevated VLCFA levels, which was compatible with the diagnosis of X-ALD. His VLCFA levels were as follows: C22:0 of 20.02, C24:0 of 33.61, C26:0 of 1.2, C24 and C22 of 1.679, and C26/C22 of 0.06. Consequently, he was started on anti-seizure medications N-acetyl-L-cysteine, and was continued on corticosteroids. He was also referred for bone marrow transplant evaluation.

## Discussion

Our patient and, most likely his deceased uncle and four brothers had X-ALD. This disorder was probably the cause of their various constitutional symptoms and the unexpected death of his male relatives. X-ALD causes progressive damage of the white matter of the parieto-occipital lobes. It also leads to behavioral problems, motor disabilities, ataxia, hearing deficit, vision loss, seizures, dementia, vegetative state and death within the first 20 years of life [1]. The diagnosis of X-ALD is confirmed by analyzing the plasma levels of VLCFAs and identifying aberrant mutations in the ABCD1 gene [1,2].

Hematopoietic stem cell bone marrow transplant is the treatment of choice for patients with cerebral X-ALD when performed at an early stage and in order to ameliorate neurological sequelea [2,5,6]. Dietary therapy, VLCFA restriction, the use of levostatin, simvastatin and anti-oxidants such as N-acetyl-L-cysteine may have a beneficial effect especially when given in early childhood to prevent further neurologic damage [6-9]. Lorenzo's oil was ineffective in cerebral inflammatory disease variants but asymptomatic patients without cerebral involvement and female carriers may potentially benefit from the early intake of oleic and erucic acids [6].

X-ALD is an uncommon cause of adrenal insufficiency [3]. In a series of 103 children with primary adrenal insufficiency diagnosed over a period of 20 years, only 15% were found to have X-ALD, various syndromes, or other idiopathic causes of adrenal insufficiency [3]. This serious endocrine gland dysfunction can be life-threatening especially in the face of stress. In patients with adrenal insufficiency, treatment with high doses of hydrocortisone is recommended for stressful events such as a major surgery or sepsis. Before surgery, a dose of 50 mg/m^2 ^of intravenous hydrocortisone 30 to 60 minutes before the induction of anesthesia, and a dose of 50 mg/m^2 ^divided every 6 hours over the next 24 hours, can be given. Further high oral or parenteral stress doses of hydrocortisone can be continued until the patient recovers [3].

Skin and mucous membrane hyperpigmentation may occur due to the elevation of proopiomelanocortin and melanocyte-stimulating hormones [3]. The usual pattern of pigmentation in adrenal insufficiency is more evident in sun-exposed regions, in areas exposed to chronic friction or pressure, in the palmar creases, and in normally pigmented areas [10,11]. Oral mucosal hyperpigmentation is considered pathognomonic of adrenal insufficiency. The lesions tend to be blue, black or brown macules in a streaky or spotted fashion [10,11]. The uniform and unusual discoloration of the lips and mucosal surfaces seen in our patient was not typical of adrenal deficiency, which might have contributed to the delayed diagnosis of his underlying neurologic and endocrine disorders.

CPAM is a space-occupying lesion composed of various types of hamartomatous anomalies believed to occur as a result of abnormal branching of immature airways during early lung development [4,12,13]. It is the most common congenital cystic lung disease and is believed to result from insults to the developing lung at 5 to 22 weeks of gestation. Three histopathological types were originally described by Stocker while two more subtypes were added by other authors [4,13].

CPAM, especially type II, is associated with other congenital anomalies such as renal agenesis, cardiac anomalies, pulmonary hypoplasia, pectus excavatum, and anasarca [13]. A majority of cases (83%) present in early post-natal period with respiratory distress, and generalized edema [13]. Some surgeons recommend conservative follow-up for asymptomatic cases but most authors recommend the surgical removal of the CPAM to treat respiratory distress and to prevent infections and cancerous transformations since up to 8.6% of primary lung tumors in the first two decades of life were associated with CPAM and lung cysts [4]. CPAM can be safely removed with excellent prognosis and virtually no morbidity or mortality occurs especially in centers with adequate experience in lung resection [4].

The association of CPAM and Addison's disease or X-ALD seen in our patient has not been described in the past. We do not know if the development of CPAM was related to prenatal adrenal insufficiency and low corticosteroid concentrations in this child. However, normal adrenocortical function is most likely important for the growth and maturation of lung tissues during the early intrauterine life [14]. Few articles reported interesting relationships between adrenocortical function and respiratory disorders in the pediatric age group. The prenatal maternal use of steroids may lead to accelerated lung maturation and reduction in the size of hamartomatous lesions [4]. At least two authors described either the resolution of hydrops fetalis secondary to CPAM or the reduction in the size of CPAMs with prenatal maternal betamethasone or dexamethasone therapy [4].

## Conclusion

We described a 3-year-old boy with X-ALD, adrenal insufficiency and CPAM. The early and accurate diagnosis and treatment of patients with X-ALD and its complications can delay progressive degenerative changes and attenuate further neurologic and metabolic dysfunction. The surgical removal of CPAM is recommended by most authors to treat respiratory distress and to prevent infections and malignant transformation. This is an original case report that may be of particular interest to pediatricians in general and of special importance to certain subspecialists such as pediatric endocrinologists, pulmonologists and neurologists.

## Abbreviations

ACTH: adrenocorticotropic hormone; BUN: blood urea nitrogen; CPAM: congenital pulmonary adenomatoid malformation; Cr: creatinine; CT: computed tomography; MRI: magnetic resonance imaging; VLCFA: very long chain fatty acids; X-ALD: X-linked adrenoleukodystrophy.

## Consent

Written informed consent was obtained from the patient's father for publication of this case report and any accompanying images. A copy of the written consent is available for review by the Editor-in-Chief of this journal.

## Competing interests

The authors declare that they have no competing interests.

## Authors' contributions

IA treated the patient and analyzed and interpreted investigations and radiologic images including the chest films and computed tomography scans. He was also a major contributor in the writing of this manuscript. SS treated the patient under the supervision of the pediatric consultants and contributed to the writing of the case report. NC treated the patient, analyzed and interpreted his metabolic investigations, and contributed to the writing of this case report. All authors read and approved the final manuscript
